# A multi-objective optimized OLSR routing protocol

**DOI:** 10.1371/journal.pone.0301842

**Published:** 2024-04-26

**Authors:** Wenhong Wei, Huijia Wu, Ying He, Qingxia Li

**Affiliations:** 1 School of Computer Science and Technology, Dongguan University of Technology, Dongguan, China; 2 School of Computer, Neusoft Institute Guangdong, Foshan, China; 3 School of Artificial Intelligence, Dongguan City University, Dongguan, China; Vellore Institute of Technology: VIT University, INDIA

## Abstract

The rapid development of mobile communication devices has brought challenges to wireless networks, where data packets are able to organize and maintain local area networks more freely without the constraints of wired devices. Scholars have developed diverse network protocols on how to ensure data transmission while maintaining its self-organizational nature. However, it is difficult for traditional network protocols to meet the needs of increasingly complex networks. In order to solve the problem that the better node set may not be selected when selecting the node set responsible for forwarding in the traditional OLSR protocol, a multi-objective optimized OLSR algorithm is proposed in this paper, which incorporating a new MPR mechanism and an improved NSGA-II algorithm. In the process of route discovery, the intermediate nodes responsible for forwarding packets are determined by the new MPR mechanism, and then the main parameters in the OLSR protocol are provided by the multi-objective optimization algorithm. Matlab was used to build a self-organizing network in this study. In addition, the conventional OLSR protocol, NSGA-II algorithm and multi-objective simulated annealing algorithm are selected to compare with the proposed algorithm. Simulation results show that the proposed algorithm can effectively reduce packet loss and end-to-end delay while obtaining better results in HV and Spacing, two multi-objective optimization result evaluation metrics.

## 1 Introduction

Ad hoc network is an instant network that does not depend on a fixed infrastructure and network topology. The nodes in such networks are able to play the role of routers and participate in data transmission [1]. With the rapid development of communication technology, Ad hoc networks are widely used in the fields of surveillance, vehicle communication and agriculture [2, 3]. Due to the dynamic nature of Ad hoc networks [4], previous researchers have developed various routing protocols suitable for different network requirements. Among them, heuristic algorithms play an important role in providing reliable solutions to real network problems because of its advantages of global optimization, strong versatility and parallelism [5].

For different activation mechanism of routing update, on-demand routing protocol, table-driven routing protocol and hybrid routing protocol are proposed [6]. The nodes in on-demand routing protocols do not need to maintain the topology information in real time, and perform route discovery operations only when nodes need to send data packets [7]. Common on-demand routing protocols include AODV (Ad hoc On-Demand Distance Vector Routing) protocols, DSR(Dynamic Source Routing) protocols, etc., table-driven routing protocols are active routing protocols. Nodes in the network maintain the routing information table in real time by exchanging information periodically [8]. Common table-driven routing protocols include DSDV (Destination-Sequenced Distance Vector Routing) protocol, OLSR (Optimized Link State Routing) protocol, etc.

This paper studies on the improvement of OLSR protocol. We not only note that the working mechanism of the OLSR protocol can still be adapted, but also take into account the optimization of heuristic algorithm. In addition, QoS (Quality of Service) has been one of the main topics of networks, it represents the quality of data transmission in a network to evaluate its performance. Hence, this paper finds an optimal node selection strategy and combines a modified heuristic algorithm to optimize the OLSR protocol. This algorithm uses QoS metrics to evaluate its performance, which can better reflect its improvement of network efficiency and resource saving.

The rest of this paper is organized as follows: Section 2 represents a brief review of related works on the optimization of Ad hoc network. In section 3, we briefly introduce the OLSR protocol, MPR mechanism and its improved algorithm. Section 4 introduces NSGA-II and the optimized mutation operator. Section 5 shows the multi-objective optimization OLSR algorithm proposed in this study. Simulation experiments and data analysis are performed in Section 6. Section 7 concludes this study and discusses the future work.

## 2 Related works

In recent years, many advanced algorithms have been used to improve Ad hoc networks. Zhang et al. used an energy-balanced routing method FAF-EBRM based on forward-aware factor in the WSN (Wireless sensor network) [9], the experimental results showed that this method guaranteed high QoS of WSN. Liu proposed a novel network partition & distance based unequal clustering routing protocol for WSN, which efficiently reduced the energy consumption and prolonged the network lifetime [10]. Zhang et al. proposed a multi-strategy channel allocation algorithm for edge computing [11], which improved the network interference, transmission delay and network throughput for Wireless mesh networks (WMNs). Zhang et al. designed a low duty cycle asynchronous MAC protocol with adaptive update mechanism based on the predicted time for WSN [12], the experimental results showed that the improved protocol can save the network energy consumption and improve the ability of the network markedly. Zhang et al. proposed a new constructing approach for a weighted topology of WSNs based on local-world theory for the Internet of Things [13], experiment results showed that the topology had robustness and fault tolerance.

Chen et al. introduced an incentive approach of flow offset based on Q-learning algorithm for IoT (Internet of Things) user privacy protection [14]. Wang et al. proposed a new approach of ID mapping correlation for radio frequency identification technology anti-collision to solve the problem of low tag recognition efficiency in IoT [15]. Zhang et al. presented a new adaptive stratified sampling based edge computing architecture [16], this architecture performed well in the real-time data stream processing of IoT. Cui et al. introduced an evolutionary game algorithm based on reinforcement learning to solve the multiple IoT devices computation offloading problem in mobile edge computing [17], the experimental results showed that the average delay of IoT devices gain good performance in dynamic environment. Chen et al. designed a new method of the IoT user perceptual task offloading grounded on quantum behavior particle population optimization strategy [18], which reduced the time consumption and energy loss of the algorithm markedly.

In [19–22], some reliable self-adaptive routing protocols based on heuristic multi-objective algorithms were presented, these methods achieved good performance. In [23], a dynamic task offloading scheme based on deep reinforcement learning was proposed, the simulation results showed that this algorithm had better performance on delay and energy consumption. In [24], a content distribution method of IOV (Internet of Vehicles) based on edge cache and immune cloning strategy was proposed to better reduce the delay of content distribution and the communication cost. Chen and Mao used a cooperative communication strategy to increase the capacity of vehicular networks [25]. In [26], a non-dominated sorting genetic strategy was proposed to solve the constrained multi-objective optimization problem for IoV in 5G, the experimental results showed that the proposed strategy can make optimal decision in actual applications. In [27], a vehicle cooperative communication method based on fuzzy logic and signal game was proposed to improve the performance of average power consumption, task success rate and invalid packet quantity for VANET. In [28], an edge caching approach based on multi-agent deep reinforcement learning was introduced to solve the problem of excessive response delay in IoV. In [29], a novel approach based on V2I direct transmission and V2V auxiliary transmission was proposed for IoV, which showed better performance in transmission delay and packet delivery rate. In [30], a passive multi-hop clustering algorithm was introduced to improve the stability and reliability of clustering algorithm for VANET (Vehicular ad hoc network). In [31], two decision algorithms were proposed to address the issue of message consistency caused by malicious vehicles that would tamper the content of disseminated messages, and the simulation results verified the effectiveness of the algorithms. In [32], a complete dataset with real traffic data was applied in a traffic simulator and was used for dynamic routing algorithms. This work provided a visualization sample of dynamic routing algorithms for Computer Science education. In [33], a new shared-bike demand forecasting model based on dynamic convolutional neural networks was proposed to predict the demand of shared bike, which achieved a high prediction accuracy within a relatively short period of time. In [34], a prefix-projection-based trajectory prediction algorithm called PrefixTP was designed to overcome the difficulty of predicting short-term partial trajectories. The experimental results showed that PrefixTP outperformed other algorithms in predicting trajectories.

Zhang et al. presented a new algorithm of clustering AODV based on edge computing strategy in IOV [35]. Experiments showed that this algorithm can reduce end-to-end delay and improve the packet delivery rate in different environments. Liu et al. proposed an adaptive repair algorithm for Temporally Ordered Routing Algorithms (TORA) routing protocol based on flood control strategy [36], which made the improved TORA more adaptable to the data transmission of the disaster relief network. Zhang et al. introduced an Ad hoc on-demand multi-path distance vector (AOMDV) routing protocol based on link lifetime and energy consumption prediction for mobile edge computing [37]. The experimental results proved that this algorithm can improve the network lifetime, reduce the node’s energy consumption and the average end-to-end delay. In [38], a network anomaly detection model using the deep learning method was proposed. By combining 1-D CNN architecture and SMOTE over-sampling method, the proposed method achieved higher accuracy rate in classifying minority classes of attacks. Zhang et al. proposed a new greedy forwarding improvement routing method for mobile ad hoc network [39]. Comparing with other existing methods, the proposed algorithm effectively reduced the network delay and prolonged the network lifetime. Liu et al. proposed an estimation formula to provide a general framework or studying the shortest path of MANET [40], which could describe the changes in actual scenes more accurately. Zhang et al. proposed an improved path finding algorithm based on genetic algorithm and bacterial foraging algorithm [41], which could improve the routing selection algorithm and convergence without change the complexity of DSR.

In [42], a new quantum-genetic based OLSR protocol was proposed. It used a new augmented Q-Learning algorithm to optimize the selection of MPR (multi-point relay) nodes that responsible for forwarding data. Harrag et al. used a multi-objective genetic algorithm to automate the selection process of the routing protocol parameters [43]. Experiments showed that it improved the QoS prominently. Yang et al. propose a multi-objective particle swarm optimization (MOPSO) framework to enhance the performance of OLSR in VANETs [44]. Gunasekar and Hinduja proposed Intelligent Water Drops algorithm to optimize the parameter setting in OLSR protocol, which improved the packet delivery ratio and reduce the communication cost in VANET [45]. Gautami et al. introduced the idea of combining Genetic Algorithm and Simulated Annealing to optimize the QoS of OLSR protocol to apply to VANET [46]. Joshua et al. proposed an optimization framework for routing protocols in VANETs based on a multi-objective firefly algorithm approach which depends on the use of network resources to further reflect the current system condition and adjust the arrangement between continuous network topology changes and the QoS needs [47].

### 2.1 Discussion

From the above studies, it can be seen that many heuristic algorithms were used to optimize the network protocols. The researchers have also changed the operators such as encoding, crossover and mutation to further optimize the initial algorithms. Other researchers have focused on the improvement of routing mechanisms to enable those routing protocols to cope with more complex environment changes and task requirements. However, there are still relatively few studies on integrated optimization from these two perspectives, indicating considerable room for improvement. This paper is an attempt at such an integrated approach, and the experiments prove that the proposed integrated algorithm also performs significantly.

## 3 OLSR protocol and MPR

### 3.1 OLSR protocol

The widespread adoption of Ad Hoc network has created more diverse network requirements, and the OLSR protocol is one of the solutions to these requirements [48]. The OLSR protocol works in the following steps:

1. Neighboring node discovery: A node discovers nodes directly adjacent to itself by broadcasting Hello messages, and maintains a list of neighboring nodes.
2. MPR selection: MPR nodes are elected in the network according to specific election rules.
3. Topology building: After the MPR nodes are selected, each MPR node establishes a table of MPR Selectors. This table indicates the nodes from which the MPR nodes are to forward the TC messages when the topology is built
4. Routing establishment and maintenance: The information in OLSR is updated periodically. If a node is found to be lost, the neighbor information is updated first, and then updated by TC grouping according to the above process. A new topology is created in the network immediately.

In summary, OLSR protocol is able to reduce the size of control packets by proactively providing paths immediately when needed and by using only selected multi-point relay nodes to forward messages [49]. Therefore, the protocol can significantly reduce network flooding and retransmissions in broadcasts. At the same time, the OLSR protocol retains the routes of all destination nodes to cope with large-scale node exchanges, making it suitable for large and dense networks [50]. The control messages of this protocol do not rely on reliable transmission, but on periodic delivery by nodes, so it can cope with a loss of some packets occasionally [51, 52].

### 3.2 MPR selection

MPR(multi-point relay) mechanism is the core of OLSR protocol. Based on this mechanism, all nodes in the network can receive messages, however, only a few selected nodes have the permission to disseminate packets. These selected nodes are called MPR nodes. The selection of MPR nodes is related to the broadcast of link state information, and is also an important step in optimizing network resource allocation. This technique can significantly reduce the number of retransmissions required to send messages to all nodes in the network [53]. The selection process of MPR nodes is as follows:

1. Choose the source node, find the set of one-hop nodes adjacent to the source node and the set of strict two-hop nodes connected to the one-hop nodes.
2. Find all the isolated two-hop neighbor nodes, set the one-hop nodes connected with them as MPR nodes, and then move the MPR nodes and all their connected two-hop nodes out of the respective sets.
3. The MPR nodes are gradually selected in descending order according to the number of connected two-hop nodes until all the two-hop nodes can be covered.

**Fig 1:**
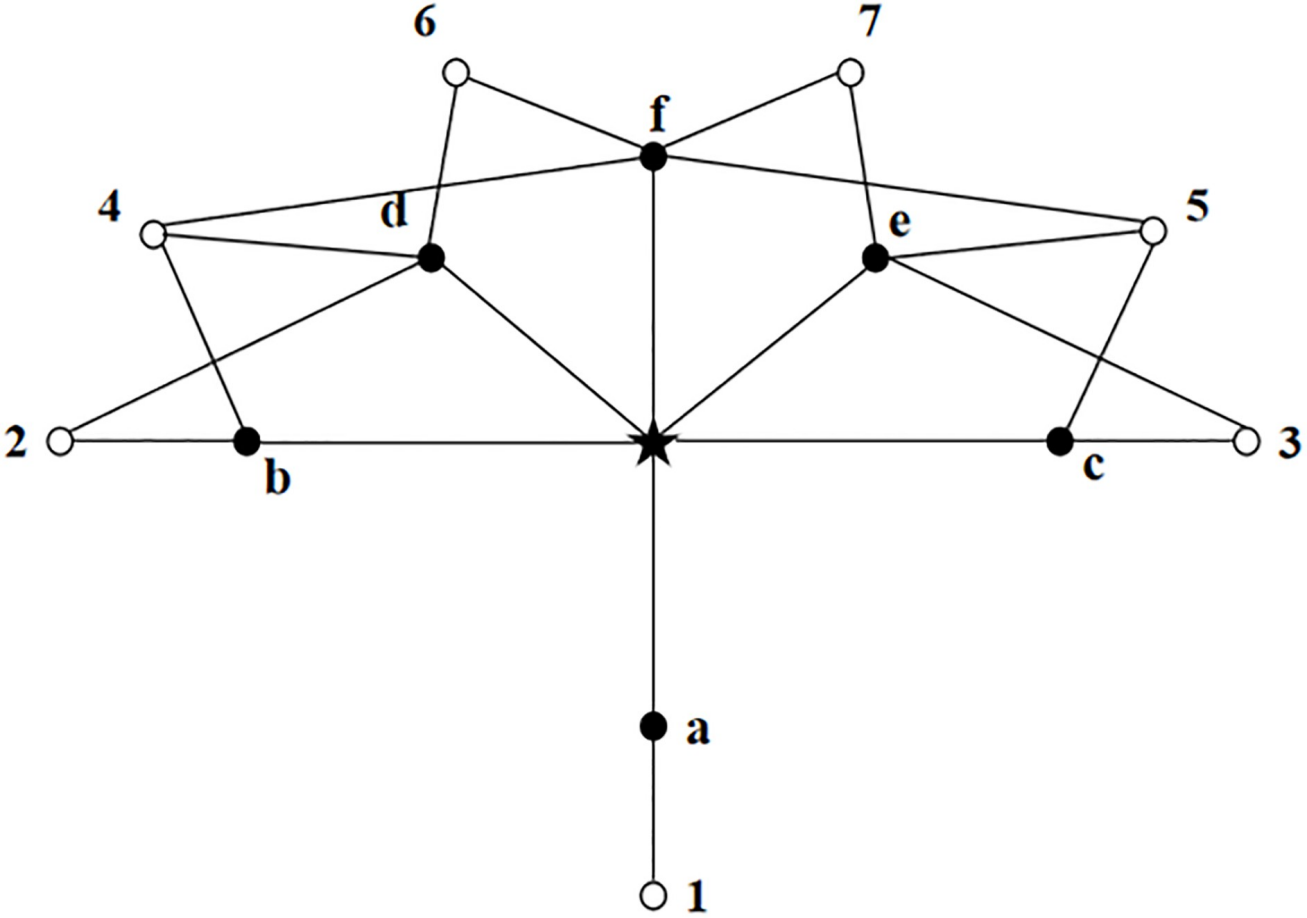
Topology diagram.

As shown in Fig 1, the star symbol represents the source node, the solid symbol represents the one-hop node, and the hollow symbol represents strict two-hop nodes. According to the above selection mechanism, the MPR set may be obtained as {a, f, b, c}, {a, f, b, e}, {a, f, d, c} or {a, f, d, e}. Each set contains four nodes.

The selection of MPR sets can speeding up the response to topology changes by reducing the maximum time interval for periodic control message transmissions, thereby improving network quality of service. The larger and denser the network, the better the optimization effect of the MPR mechanism.

### 3.3 Improved MPR scheme

However, in some cases, the MPR set selected by the traditional OLSR protocol is not the optimal set, which will lead to unnecessary packet forwarding and waste of network resources [54]. Based on previous research, Dong and Zhang [55] proposed a reverse MPR set selection scheme, which can optimize resource allocation, and improve data transmission efficiency through loop and set operations. The process of the mechanism is as follows:

(1) Find the one-hop neighbor set F and the two-hop neighbor set S in the topology, and initialize the MPR set.
(2) Map each one-hop neighbor to its connected strict two-hop neighbor one by one.
(3) Sort the set F from largest to smallest according to the number of two-hop neighbors that each one-hop neighbor can connect. If the numbers are the same, then sort the nodes alphabetically.
(4) Find the isolated two-hop node in set S, put the corresponding one-hop node into the MPR set, and mark these two nodes. If there is no such one-hop node, delete the lowest-ranked node in the sorted set F, and perform a union operation on all two-hop nodes that connected to each node in the set F. Compare whether the union is consistent with the set of unmarked nodes in the set S. If so, it can be confirmed to be deleted. Otherwise, it is added to the MPR set, and its connected two-hop node is marked.
(5) Repeat step (4). The union is performed every time a one-hop node moves to the MPR set until the elements in the MPR set can completely cover the set S.

Applying this scheme to Fig 1 again, the new MPR set can be obtained is {a, d, e}, which has one node less than the original MPR set.

## 4 Genetic algorithm and its improvement

### 4.1 MOGA

Genetic Algorithm (GA) is a computational model that simulates the natural selection and genetic mechanism of Darwin’s theory of biological evolution [56]. The model adopts a probabilistic optimization method, which can automatically obtain and guide the optimized search space without certain rules, and adjust the search direction adaptively. This algorithm takes all the individuals in the population as objects and uses randomization techniques to perform an efficient search of a parameter space that is encoded [57]. The genetic operation includes selection, crossover, and mutation.

Multi-objective optimization means that when multiple objectives need to be reached in a given scenario [58], the optimization of one objective is at the expense of the deterioration of the others due to the tendency to have inherent conflicts between the objectives. In this case, it is difficult to emerge a unique optimal solution, and instead, coordination and compromises are made among them so that the overall objective is as optimal as possible [59]. The result of optimization is a set of optimal solutions obtained on the Pareto front.

Multi-objective evolutionary algorithm is suitable for solving complex multi-objective optimization problems and has been widely used. This algorithm uses one-dimensional string data to represent variables, also known as genotyped individuals. A certain number of individuals form a population, which will undergo genetic recombination -> mutation -> evaluation and selection to produce new individuals [60]. Individuals with better fitness will be remained in the next generation, that is, the non-dominant solution.

This study adopts the framework of the non-dominated sorting genetic algorithm with elite strategy (NSGA-II), which introduces fast non-dominated sorting and crowding distance comparison operator. Individuals can judge the superiority of each other by comparing the rank and crowding distance, which has the advantages of simple and efficient computation.

### 4.2 Precision control mutation operator

Zhang et al. [61] proposed an adaptive precision control mutation operator to explore and exploit the decision space. Let *x* = [*x*_1_, *x*_2_, …, *x*_*N*_]^*T*^ represents one individual, *x*_*i*_ represents the *i*th decision variable of individual *x*. Eqs (1)–(6) are used to explore the local region near *x* and the region far away from *x*

$$x_{i}^{\prime }=x_{i}+\Delta \alpha$$ (1)

$$x_{i}^{\prime }=x_{i}-\Delta \alpha$$ (2)

where,

$$\Delta \alpha =\frac{1}{10^{\text{Random}\left(\text{p}\right)}}\times \text{Random}\left(9\right)$$ (3)

$$x_{i}^{\prime }=x_{i}+\Delta \beta$$ (4)

$$x_{i}^{\prime }=x_{i}-\Delta \beta$$ (5)

where,

$$\Delta \beta =\frac{1}{10^{\text{Random}\left(\text{q}\right)}}\times \text{Random}\left(9\right)$$ (6)

Eqs (1) and (2) represent the increase or decrease of *x*_*i*_ by Δ*α*, Δ*α* is calculated by Eq (3). The function Random(*p*) is used to generate a pseudo-random number between 1 and *p*. $\frac{1}{10^{\text{Random}\left(\text{p}\right)}}$ is used to control the required local search accuracy, Random(9) produces a random coefficient ranging from 1 to 9. These two equation are used to perform local exploitation. If a local search accuracy of 0.0001 is desired, the parameter *p* should be set to 4. Eqs (4) and (5) represent the increase or decrease of *x*_*i*_ by Δ*β*, Δ*β* is calculated by Eq (6). The function Random(*q*) is used to generate a pseudo-random number between 1 and *q*. $\frac{1}{10^{\text{Random}\left(\text{q}\right)}}$ is used to control the required global search accuracy. These two equation are used to perform global exploration.

## 5 Multi-objective optimized OLSR protocol

### 5.1 Network model

OLSR performs basic functions through different types of control message. Among them, each node must detect its neighbor nodes with direct and bidirectional links. In order to ensure the validity of the links, each node periodically sends Hello messages to its neighbors [62]. In this process, the neighbor list in the Hello message may be partial, so it is stipulated that all neighbor nodes are referenced at least once during a predefined refresh cycle. Topology Control (TC) messages are sent periodically by each node in the network to announce its MPR node set [63], thus building an internal forwarding database. The address list in each TC message may also be incomplete, but must be resolved within a certain refresh period. Refresh interval refers to the period in which a node must refer to each link and each neighbor [64]. This value is also used to determine the neighbor maintenance time.

According to [43], Hello interval, Topology control (TC) interval and Refresh interval are taken as three variables of the multi-objective optimization algorithm in this study. In accordance with RFC 3626, these three variables are real numbers ranging from 1 to 30 and will be used as objects for selection, crossover and mutation operations during the optimization process.

### 5.2 Objective function

In this study, packet loss rate and end-to-end delay are taken as two objectives of optimization [43], both of which are important indicators for network quality evaluation [65]. The packet loss rate refers to the ratio of the number of lost packets to the sent packets during the transmission process. The end-to-end delay refers to the total delay of sending a packet from the source host to the destination host.

### 5.3 The improved OLSR

**Fig 2:**
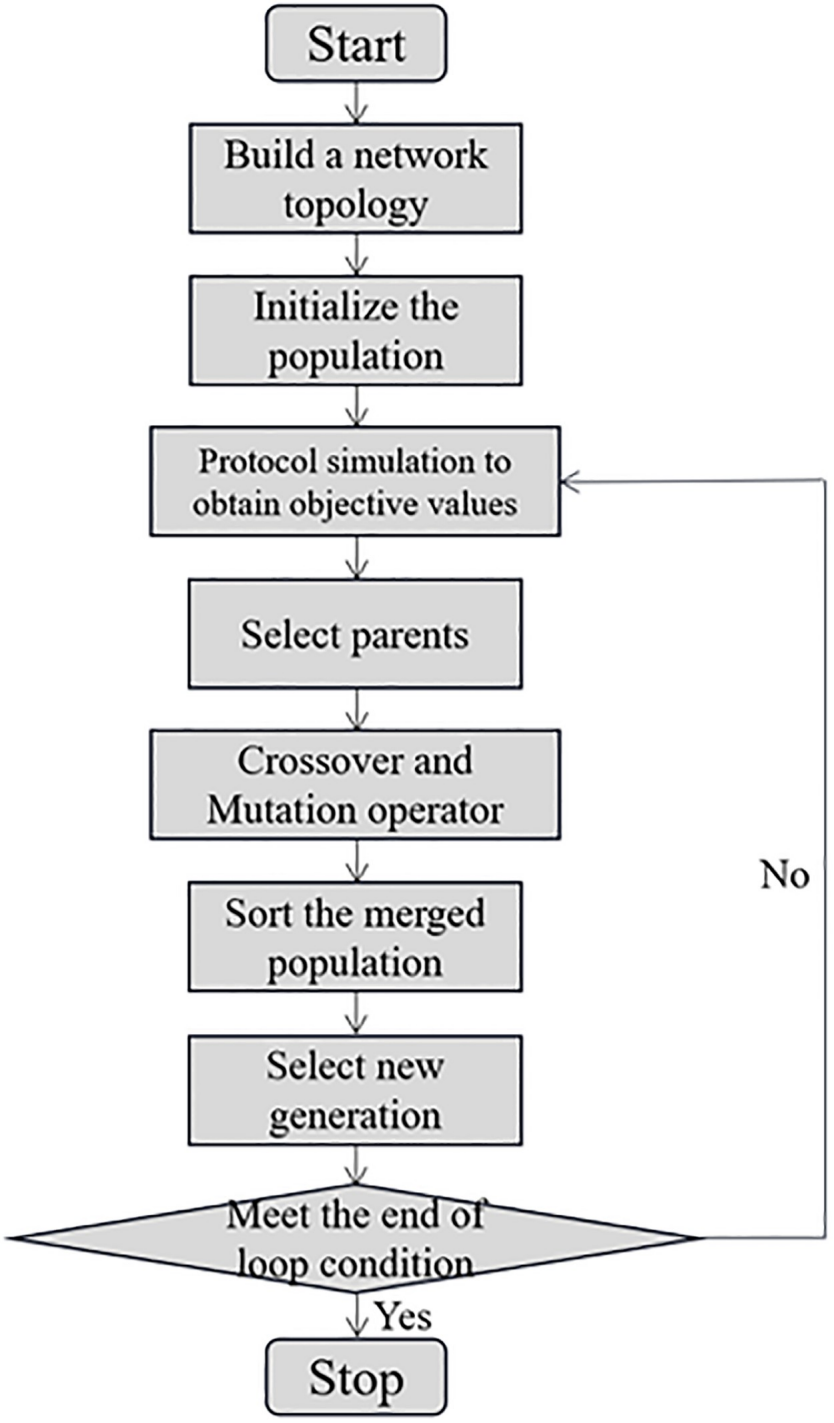
Flow chart of the proposed algorithm.

The brief workflow of the improved OLSR is as follows: First, build the network and initialize the population. Use the modified MPR mechanism to determine the routing path. Then, the objective values of the initial population is obtained by simulation experiments. Start the population iteration until the end of loop condition is met. The flow chart is shown in Fig 2.

### 5.4 Pseudo code

Improved OLSR protocol Based on new MPR Mechanism Combined with Optimized NSGA-II Algorithm

1. Build the topology, set the parameters of the network and the multi-objective optimization algorithm

2. Initialize the population, set the population size pop_size and number of iterations

3. Calculate the two objective values of each individual through network simulation, and sort each individual

4. Screening of parental individuals

5. Crossover between parent individuals to obtain offspring

6. Select individuals from the parents for mutation to obtain offspring using the mutation method in Section 4.2.

7. Merge the offspring and the original population

8. Perform a non-dominated sorting on the merged population and select a new generation of the population

9. Loop step 3 to 8 until the iteration is completed and evaluate the final results

### 5.5 Complexity analysis

The time complexity of OLSR protocol depends on the size and connectivity of the network, as well as on the implementation and optimization algorithm. Overall, the complexity of the OLSR protocol is low because it uses a multi-point relay-based routing calculation method, which can efficiently calculate the routing path of packets, and the protocol itself does not have much redundant information. Specifically, the time complexity of the OLSR protocol can reach O(*m*^3^) in the worst case, where *m* is the number of nodes in the network. However, in most cases, the time complexity of the OLSR protocol can be optimized to O(*m*^3^) or lower.

The improved OLSR protocol contains two main computational components. One is the selection of the MPR set, because the improved MPR mechanism requires needs to compute the shortest path from each node to other nodes, and for each node, it needs to find the smallest set of all its neighboring nodes, which requires *n* iterations and each iteration needs to compare the number of neighboring nodes. Therefore, the total time complexity is *O*(*n*^2^). The other is the multi-objective optimization algorithm. In this part, the computational complexity of this algorithm is related to the number of objectives *M* and the number of individuals *N*. The sorting process of selecting an individual in each round takes *O*(*N*) and has to compare *M* objectives, so the complexity here is *O*(*MN*^2^). To summarize, the total complexity of the improved OLSR protocol is *O*(*MN*^2^)+*O*(*n*^2^).

### 5.6 Discussion

Compared with the previous studies cited in Section 2, this paper also makes some improvements to the relevant strategies and algorithms for specific network environments, and uses simulation experiments to verify the effectiveness and practicality of the improved algorithms. In addition, the network quality measures such as packet loss rate and delay are also used to reflect the optimization effect on the network. In previous studies, researchers have made in-depth analysis of the research background, contributed extensively to various types of network, and their results have been quite convincing. In this paper, we choose the OLSR protocol as the research object and use the Pareto solution to deal with the multi-objective problem, and add data analysis to enhance the persuasiveness of the results.

## 6 Simulation and analysis

Unlike traditional optimization ideas, the proposed algorithm optimizes the OLSR protocol in terms of both multi-objective optimization and routing mechanism. In this study, Matlab is used as a network simulation and multi-objective optimization algorithm implementation tool. The traditional OLSR protocol, the traditional NSGA-II optimized OLSR protocol and the multi-objective simulated annealing algorithm [66] optimized OLSR protocol are selected for comparison experiments. With the same network topology, the same number of packets are sent in each experiment to calculate the average packet loss rate and the average end-to-end delay.

### 6.1 Evaluation indicators

#### 6.1.1 HyperVolume(HV)

The HV evaluation index represents the volume of the hypercube enclosed by the non-dominated individuals in the solution set and the reference point in the target space [67]. This index evaluates both the convergence and distributivity of the solution set at the same time [68]. The accuracy of HV metric depends on the choice of reference point, and different reference points for the same solution set will give different results [69]. Let *X* denote the non-dominated solution set and *P* be the corresponding reference point. The calculation formula (7) of HV is as follows

$$\text{HV}\left(\text{X},\text{P}\right)=\cup_{\text{x}\in \text{X}}^{\text{X}}\text{v}\left(\text{x},\text{P}\right)$$ (7)

*x* is an individual in the solution set *X*, i.e., *x*∈*X*. v(x, P) denotes the volume of the space enclosed by *x* and *P*. *U* is the mathematical symbol of union set. This formula represents the union of all volumes. The larger the value, the closer the solution set is to the real Pareto front, and the better the algorithm performance.

#### 6.1.2 Spacing(SP)

The SP index is used to assess the uniformity of the approximate Pareto solution over the target space [70]. Assuming that the solution set obtained by the multi-objective algorithm is *P*, the calculation formula (8) of SP is shown below

$$\text{Spacing}\left(\text{P}\right)=\sqrt{\frac{1}{\left|\text{P}\right|-1}\sum_{\text{i}=1}^{\left|\text{P}\right|}{\left(\bar{d}-d_{i}\right)}^{2}}$$ (8)

$\bar{d}$ is the average of the distance between all individuals and their nearest individual, *d*_*i*_ is the current individual. This formula measures the standard deviation of the minimum distance from each individual to the other individuals. The smaller the Spacing value is, the more even the solution set is. If the value of SP is 0, it means that the Pareto solution is uniformly distributed on the target space.

### 6.2 Experiment setting

**Fig 3:**
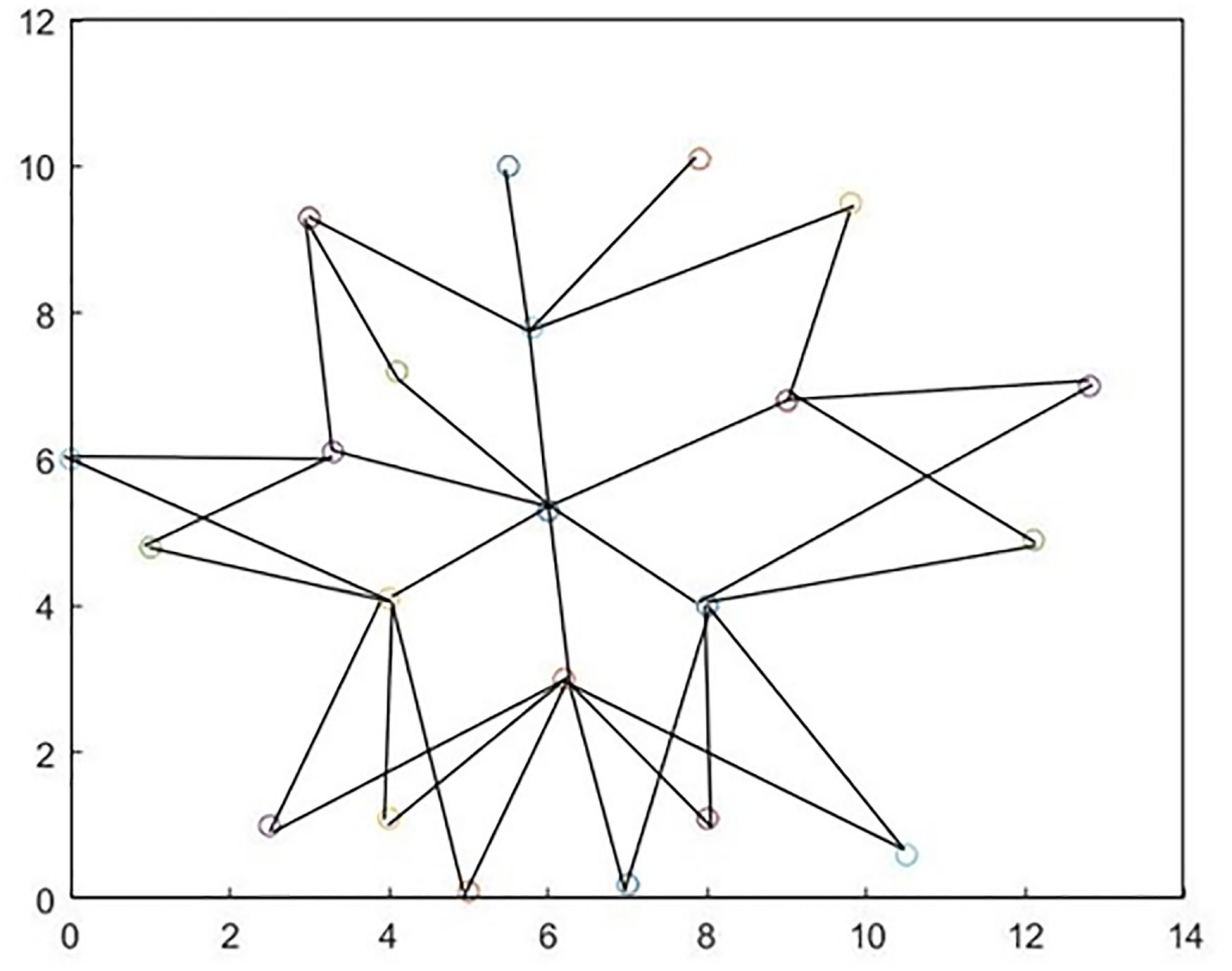
Network topology.

The network parameters and optimization algorithm parameters are shown in Table 1. The network topology is shown in Fig 3.

**Table 1:** Simulation experiment parameters.

network parameters		proposed algorithm parameters	
nodes connections packets sent	22 34 200	population targets variables crossover rate mutation rate	100 2 3 0.6 0.1

### 6.3 Simulation experiment results

**Fig 4:**
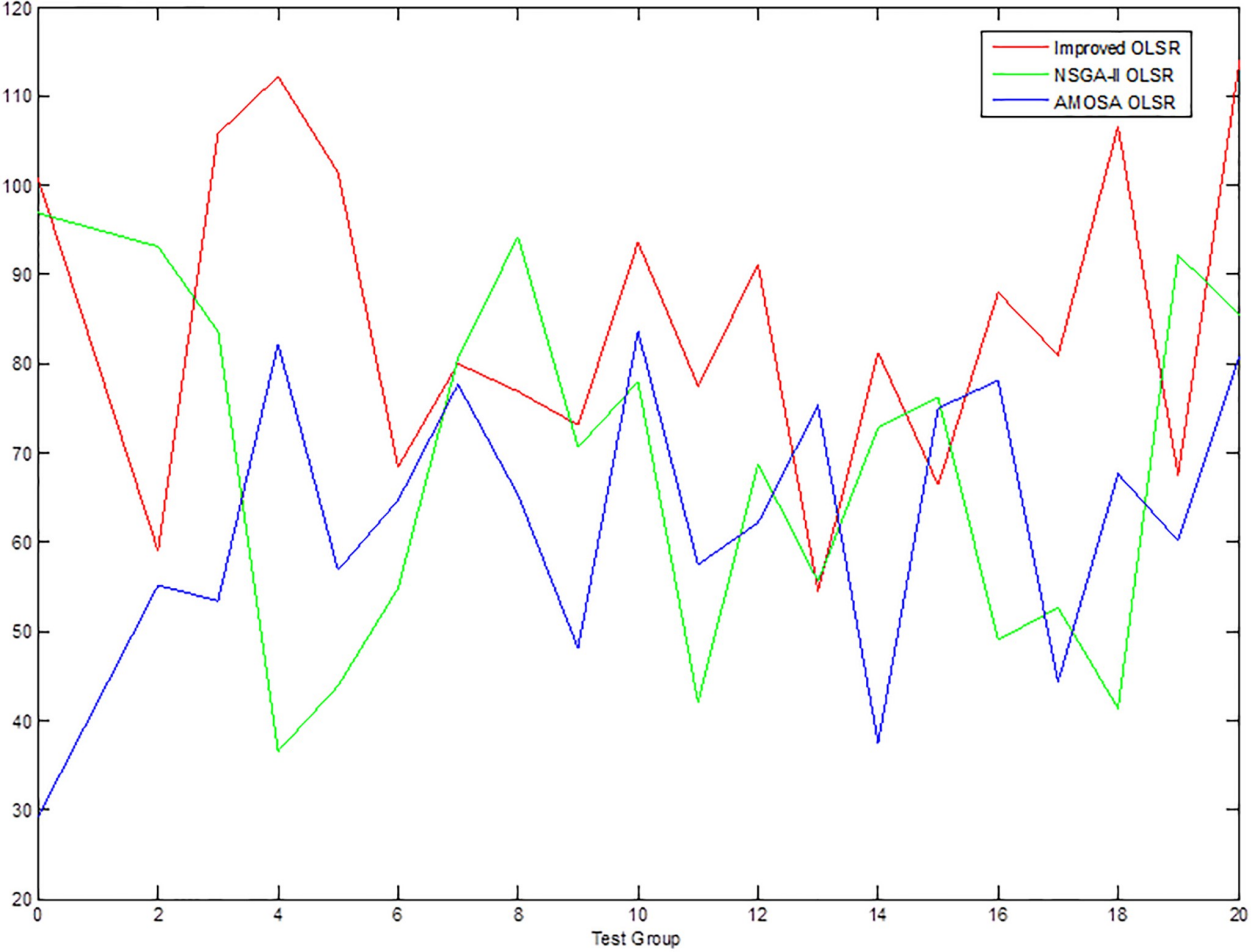
Comparison graph of HV.

**Fig 5:**
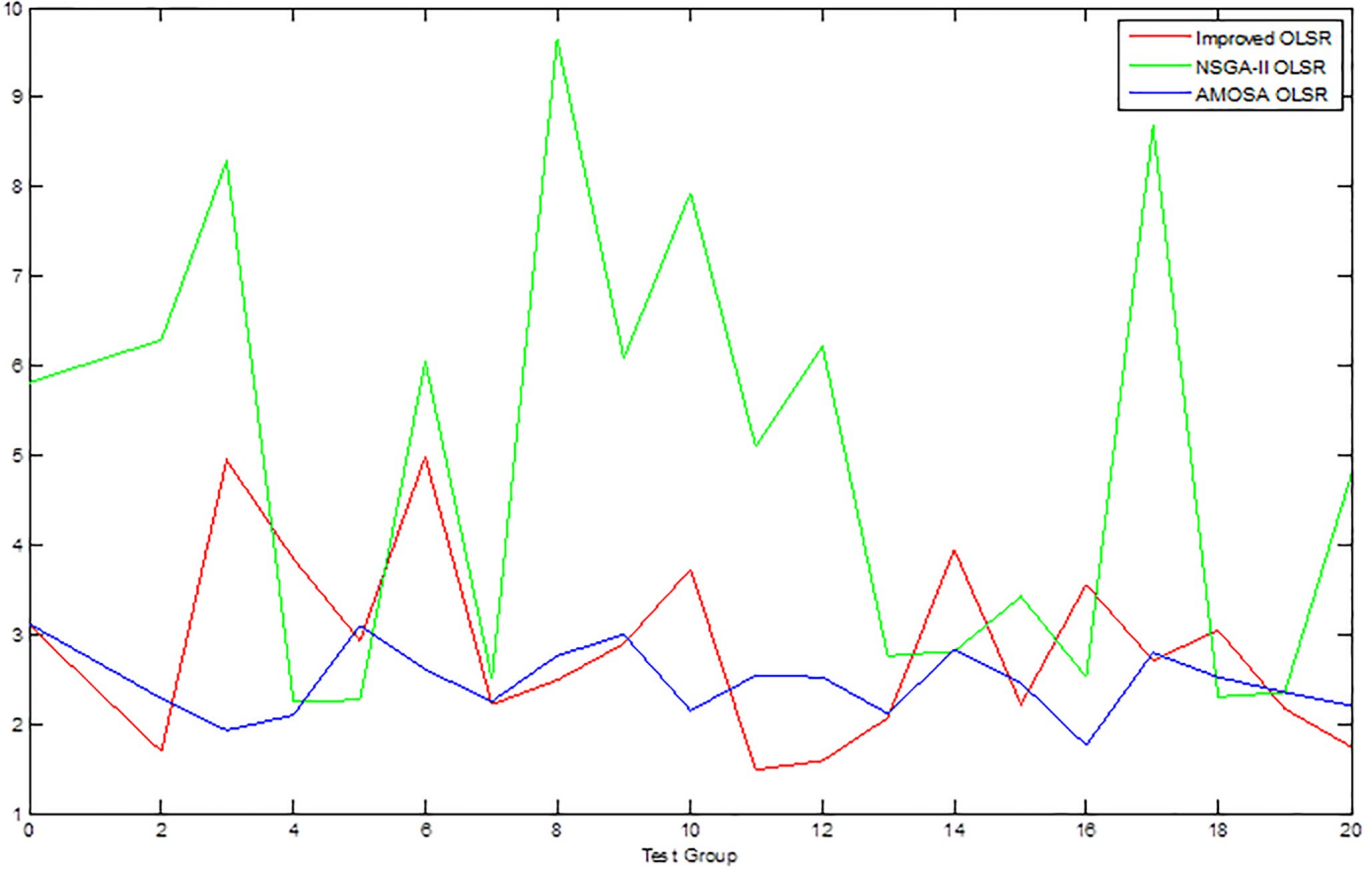
Comparison graph of SP.

**Fig 6:**
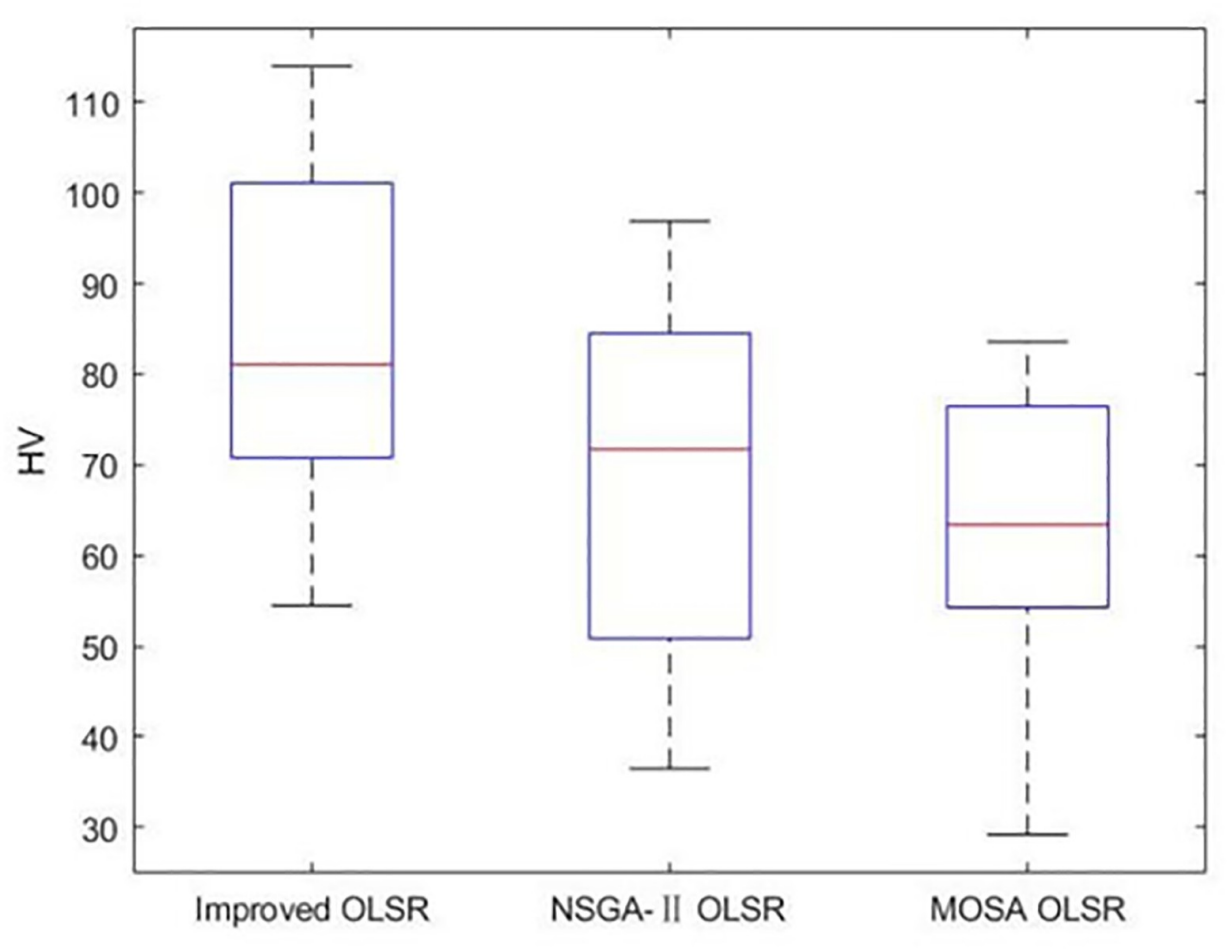
Boxplot of HV.

**Fig 7:**
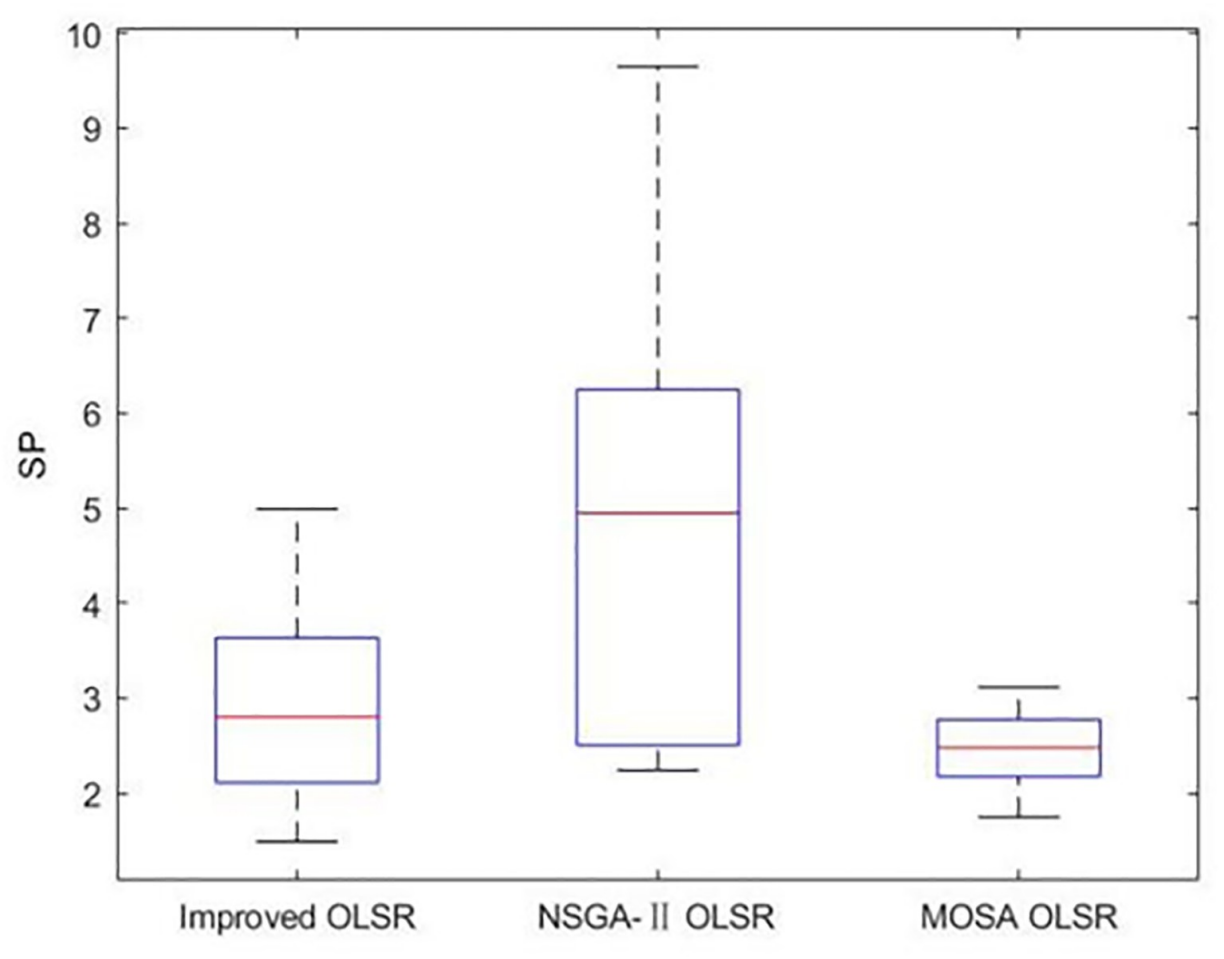
Boxplot of SP.

**Fig 8:**
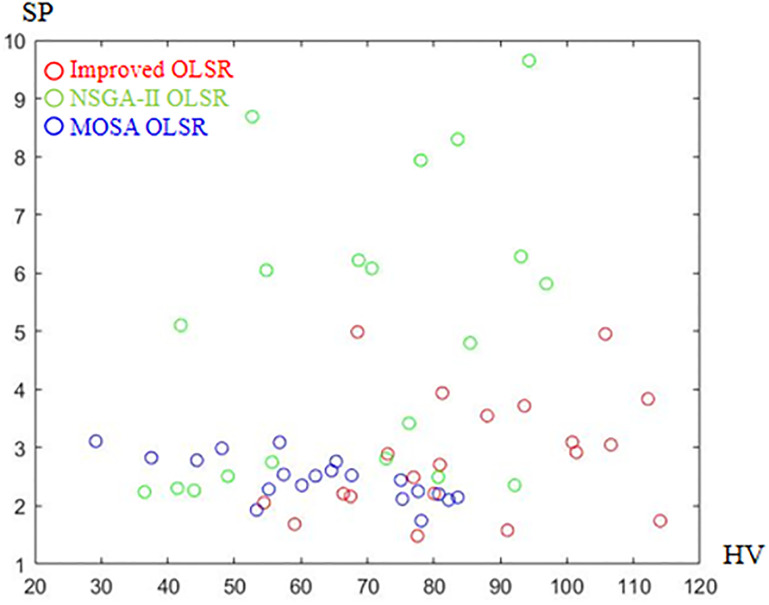
Scatter plot of HV and SP.

**Fig 9:**
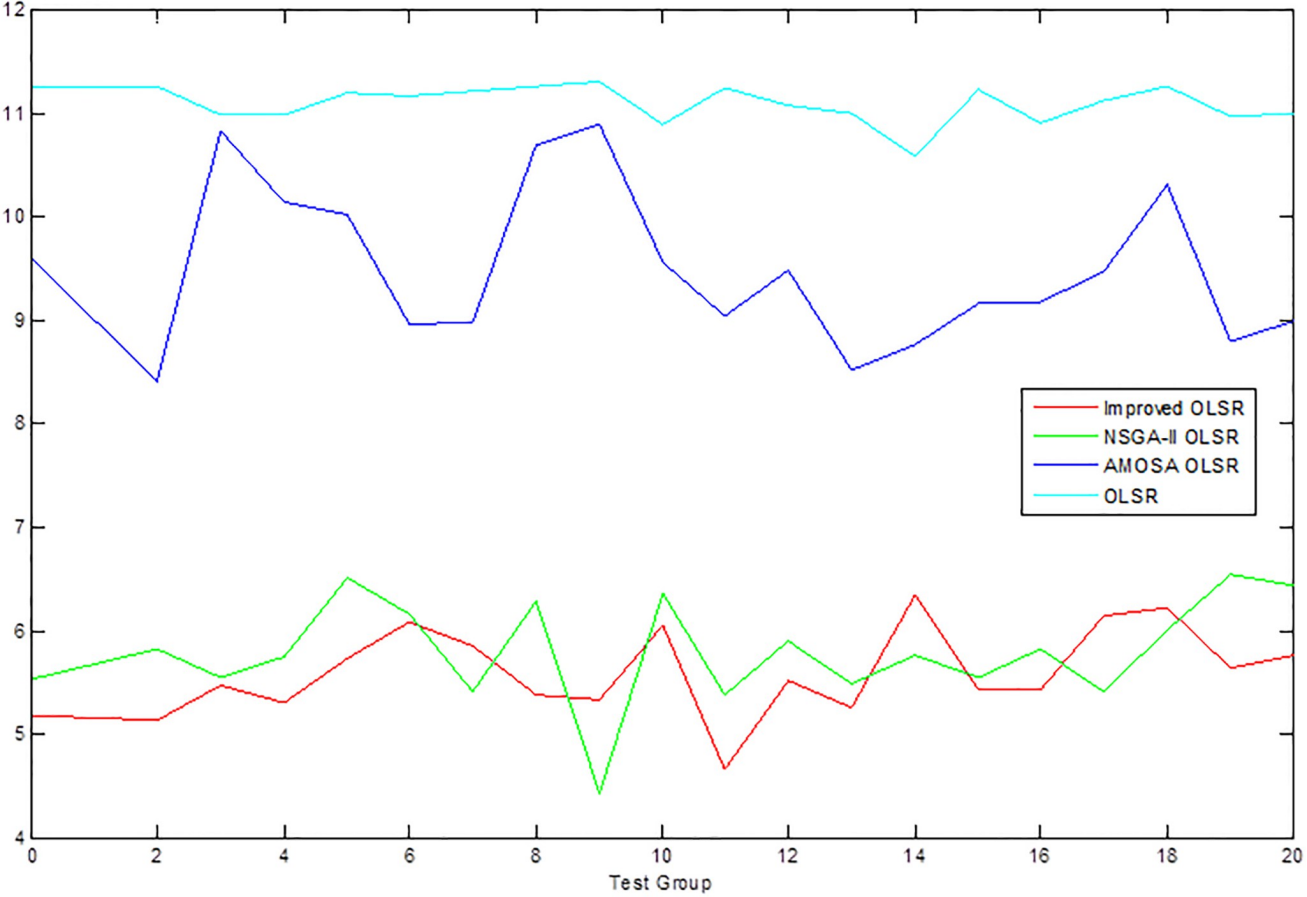
Comparison graph of packet loss rate.

**Fig 10:**
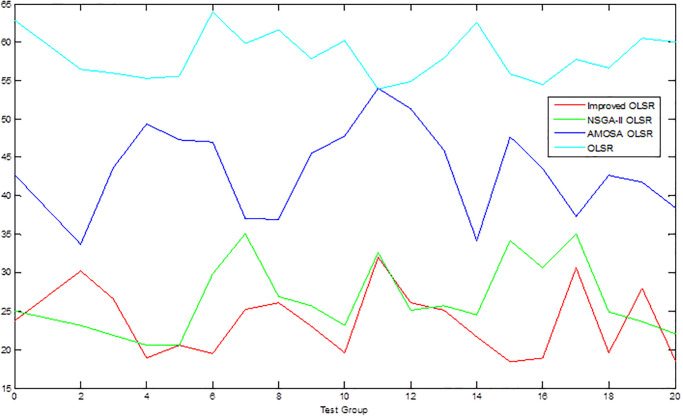
Comparison graph of end-to-end delay.

**Fig 11:**
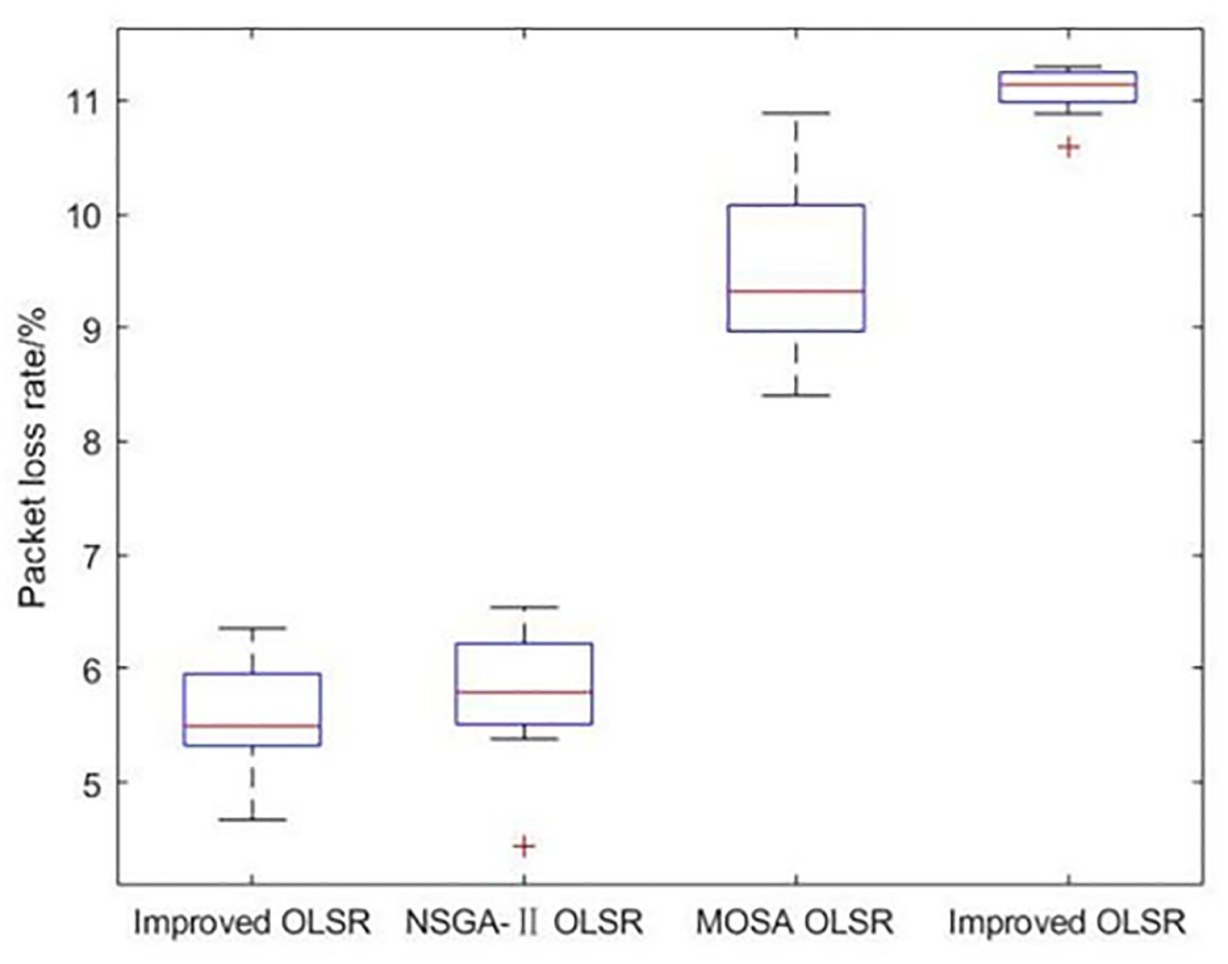
Boxplot of packet loss rate.

**Fig 12:**
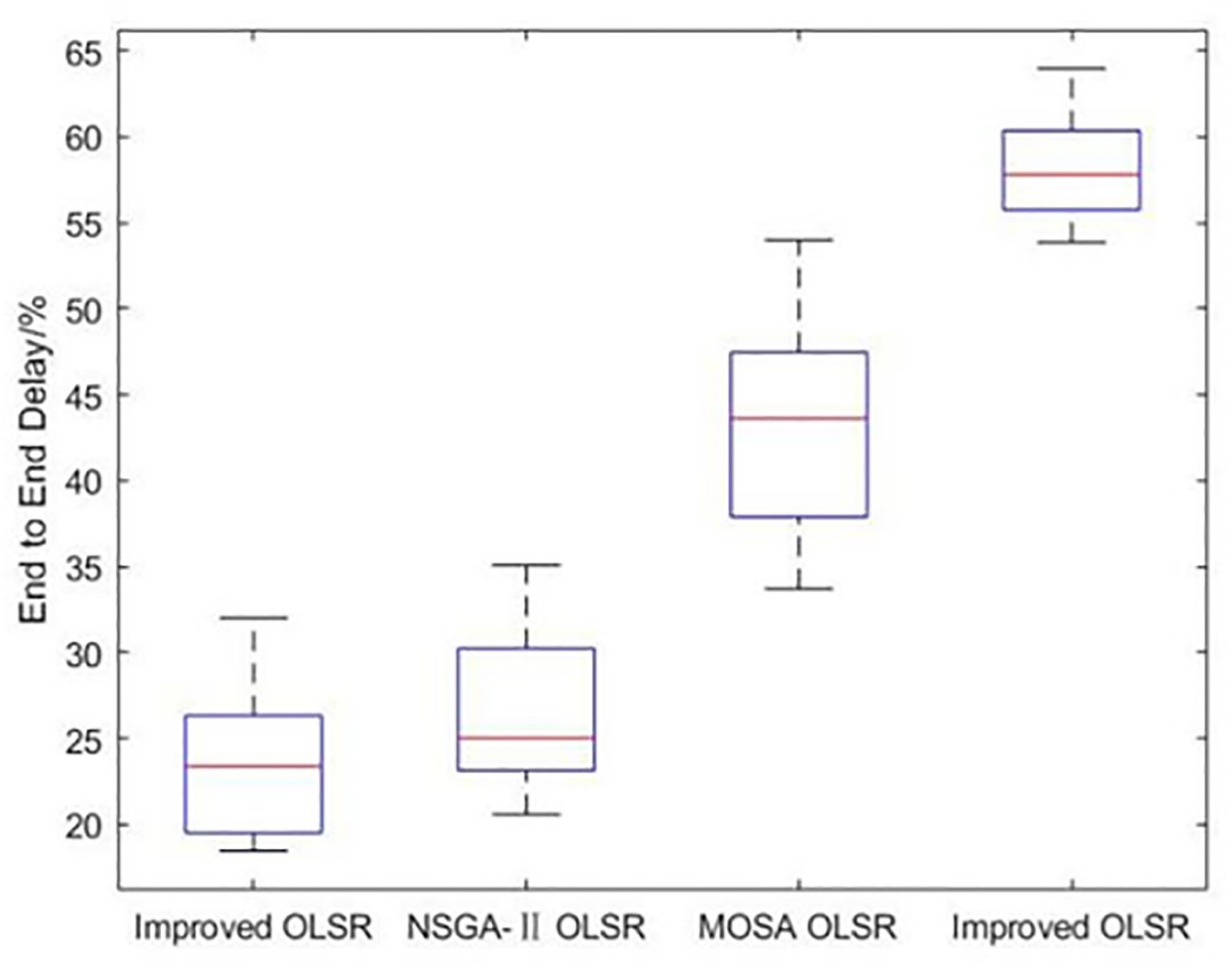
Boxplot of end to end delay.

**Fig 13:**
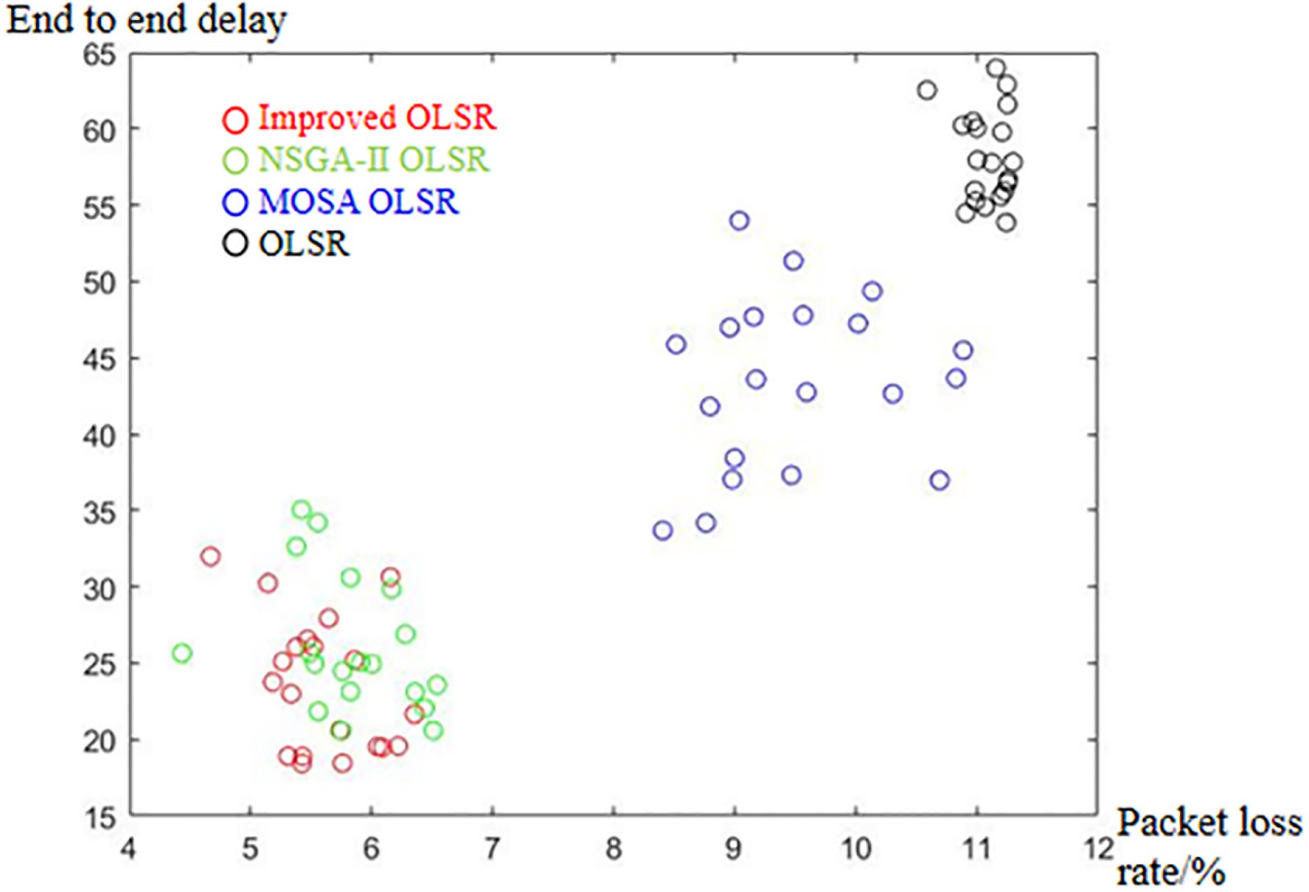
Scatter plot of packet loss rate and end to end delay.

Table 2 shows the collection of the solution set evaluation metrics obtained from all the experiments of multi-objective optimized OLSR protocol and run time for each algorithm for each run. Table 3 shows the collection of the network quality metrics obtained from all experiments of the multi-objective optimized OLSR protocol and the original OLSR protocol, where the number of packets sent in each simulation is 200, and the average packet loss rate and end-to-end delay are counted in each algorithm. Figs 4 and 5 show the data in Table 2 as bar graphs. Figs 6 and 7 show the data in Table 2 as boxplots. Fig 8 shows the data in Table 2 as scatter plot. Figs 9 and 10 show the data in Table 3 as bar graphs. Figs 11 and 12 show the data in Table 3 as boxplots. Fig 13 shows the data in Table 3 as scatter plot.

**Table 2:** Evaluation index of multi-objective optimization algorithm.

Improved OLSR			NSGA-II OLSR			AMOSA OLSR		
HV	SP	run time/s	HV	SP	run time/s	HV	SP	run time/s
**100.813254**	**3.095831**	**6.597439**	96.906755	5.816321	7.234401	29.165	3.1133	7.316922
59.05023	**1.688376**	7.031235	**93.092785**	6.285952	**6.789962**	55.167	2.2868	7.931146
**105.775363**	4.954058	**6.487442**	83.581825	8.297134	6.552379	53.342	**1.9313**	7.698046
**112.19943**	3.839177	6.785653	36.486627	2.240588	**6.18666**	82.224	**2.1031**	8.398576
**101.406916**	**2.922663**	**6.503328**	43.927112	2.266727	7.509054	56.801	3.0929	7.633853
**68.510852**	4.98929	**6.992628**	54.805031	6.046629	7.081911	64.604	**2.6054**	8.536014
80.05946	**2.212217**	7.228467	**80.665947**	2.49649	**7.14628**	77.631	2.2522	8.123185
76.934	**2.4922**	**6.662898**	**94.318344**	9.648715	7.08785	65.295	2.7649	7.544914
**73.051701**	**2.892598**	7.577904	70.671686	6.08017	**7.031838**	48.11	2.9902	7.966633
**93.60565**	3.719562	6.944842	78.042357	7.937279	**6.30737**	83.581	**2.148**	7.758269
**77.534**	**1.4871**	**6.823574**	41.935922	5.102679	7.57574	57.405	2.5397	7.496716
**91.060545**	**1.583228**	7.367366	68.686855	6.220011	**6.837195**	62.192	2.5167	8.073279
54.448	**2.0556**	7.091183	55.649806	2.75291	**6.763237**	**75.255**	2.1199	7.953578
**81.258575**	3.939789	**6.321039**	72.801959	**2.81347**	6.351201	37.503	2.8276	7.890938
66.375	**2.2114**	8.066726	**76.257826**	3.420455	**6.801208**	75.015	2.4449	7.89487
**88.019122**	3.551554	7.885388	49.037576	2.511725	**6.771911**	78.109	**1.7481**	8.219964
**80.885624**	**2.708957**	**7.673384**	52.669424	8.686493	8.696988	44.362	2.784	7.820719
**106.612632**	3.051339	**6.336094**	41.427	**2.3045**	7.725367	67.629	2.5262	7.232223
67.459	**2.1623**	**6.742672**	**92.153597**	2.356938	6.965776	60.136	2.3549	7.393517
**114.028824**	**1.746736**	**6.832979**	85.445738	4.797804	7.200151	80.732	2.2024	8.030539

**Table 3:** Network simulation results.

Improved OLSR		NSGA-II OLSR		AMOSA OLSR		OLSR	
Packet Loss/%	E2E Delay	Packet Loss/%	E2E Delay	Packet Loss/%	E2E Delay	Packet Loss/%	E2E Delay
**5.185**	**23.7641**	5.53	24.97236	9.5946	42.758	11.25	62.90444
**5.145**	30.24631	5.825	**23.1391**	8.4063	33.701	11.255	56.48142
**5.47**	26.55857	5.56	**21.8367**	10.83	43.667	10.985	55.97741
**5.31**	**18.923**	5.75	20.58363	10.138	49.371	10.99	55.27264
**5.74**	20.617	6.51	**20.591**	10.021	47.254	11.2	55.57722
**6.09**	**19.488**	6.165	29.86335	8.96	46.996	11.16	63.98024
5.86	**25.2122**	**5.42**	35.07	8.9808	37.037	11.21	59.79158
**5.38**	**26.062**	6.28	26.90672	10.694	36.973	11.255	61.6043
5.335	**22.9998**	**4.435**	25.65928	10.89	45.505	11.3	57.82639
**6.05**	**19.5466**	6.36	23.10529	9.5667	47.8	10.885	60.2346
**4.67**	**31.997**	5.38	32.65184	9.0395	53.997	11.245	53.87124
**5.52**	**26.093**	5.91	25.06542	9.4865	51.36	11.07	54.89811
**5.265**	**25.121**	5.49	25.67362	8.5172	45.889	11.005	57.96153
6.355	**21.6675**	**5.76**	24.48977	8.7632	34.195	10.59	62.53533
**5.425**	**18.442**	5.555	34.21914	9.1571	47.696	11.23	55.92694
**5.43**	**18.8952**	5.825	30.61514	9.1774	43.599	10.91	54.49625
6.155	**30.6536**	**5.42**	35.07	9.4688	37.327	11.125	57.78028
6.22	**19.582**	**6.005**	24.95618	10.307	42.661	11.26	56.69773
**5.645**	27.95686	6.54	**23.5853**	8.7963	41.818	10.97	60.47512
**5.76**	**18.4671**	6.44	22.05004	9	38.439	11	60.05679

As can be seen from Table 2 and Fig 4, most HV values of the improved OLSR optimization algorithm are larger than NSGA-II and significantly better than MOSA. This indicates that the non-dominated solution of the proposed algorithm is closer to the real Pareto front. Fig 6 shows the discrete distribution of HV values for the three algorithms, and it can be observed that the data of the proposed algorithm is generally distributed on the upper position. From Table 2 and Fig 5, most SP values of the improved OLSR optimization algorithm are better than MOSA, and significantly better than NSGA-II. This indicates that the distribution of the solutions of the proposed algorithm is more even. Fig 7 shows that the proposed algorithm can obtain lower SP values. By observing the two indicators simultaneously in Fig 8, it can be seen that the improved algorithm distributes more points in the lower right corner of the coordinate axis, which indicates that the algorithm takes more cases of two better indexes at the same time. Both the HV and SP values of the proposed algorithm outperform the other two multi-objective optimization algorithms in the 1st, 5th, 9th, 11th, 12th, 17th and 20th simulation experiments, showing the advantages of the proposed algorithm.

Comparing the run time of these three algorithms, it can be counted that the improved algorithm has the shortest run time in 11 experiments, NSGA-II OLSR has the shortest running time in 9 experiments, while AMOSA OLSR has the longest run time in each experiment. According to the analysis in Section 5.5, the proposed algorithm differs from NSGA-II OLSR in the selection of MPR and the mutation process, while these two do not have a significant difference in computing time consumption. However, AMOSA OLSR differs from the other two algorithms in the Optimization mechanism, and runs for a relatively longer time.

From Table 3 and Fig 9, it can be seen that most of the packet loss rates of the improved OLSR optimization algorithm are lower than those of NSGA-II OLSR. And, in all experiments, lower than MOSA OLSR and the original OLSR, which indicates that the proposed algorithm can effectively reduce the packet loss rate and ensure the transmission efficiency. Fig 11 shows that the packet loss rate of the proposed algorithm is generally lower than that of the other three algorithms. As can be seen from Table 3 and Fig 10, the end-to-end delay of the improved OLSR optimization algorithm is lower than that of NSGA-II OLSR in most of the experiments and lower than the other two algorithms in all experiments, which indicates that the proposed algorithm can effectively reduce the end-to-end delay. Fig 12 shows that both the quartile and median positions of the end-to-end delay values of the proposed algorithm are lower than those of the other algorithms, which further proves its superiority. In Fig 13, packet loss rate the end-to-end delay of all algorithms are scattered in the coordinate axis at the same time, and it can be seen that the proposed algorithm is mainly concentrated in the lower left corner, which indicates that it is more common for this algorithm to achieve lower packet loss rate and end-to-end delay simultaneously, and the performance of this algorithm is better. In the 1st, 4th, 6th, 8th, 10th, 11th, 12th, 13th, 15th, 16th and 20th set of experiments, both packet loss rate and end-to-end delay of the proposed algorithm are optimal, which proves that it can effectively improve network service quality.

### 6.4 Statistical analysis

In this section, Wilcoxon test and Friedman test are used to compare the above algorithms. Tables 4 and 5 show the results of Wilcoxon test between this algorithm and the two comparison algorithms. Table 4 compares the HV value, and Table 5 compares the SP value. The Wilcoxon test is used to test the null hypothesis and is able to rank the observed distribution [71]. In these four sets of comparison data, it is evident by the three metrics R+, R- and p-value that the proposed algorithm has a larger rank sum than the other two algorithms. This illustrates that the improved OLSR algorithm is significantly different from the other two algorithms in terms of HV and SP indexes.

**Table 4:** Wilcoxon test of the HV metric values.

Improved OLSR versus	R+	R-	p-value
NSGA-II	14.00	6.00	0.025094
MOSA	18.00	2.00	0.00077959

**Table 5:** Wilcoxon test of the SP metric values.

Improved OLSR versus	R+	R-	p-value
NSGA-II	15.00	5.00	0.0064246
MOSA	13.00	7.00	0.68132

**Fig 14:**
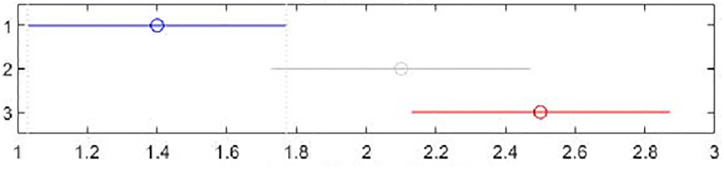
Friedman test figure of the HV metric values.

**Fig 15:**
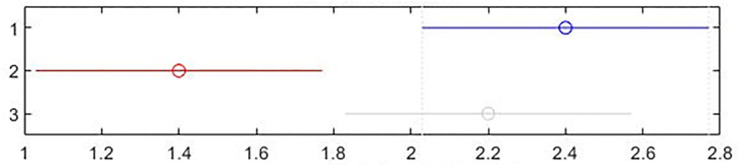
Friedman test figure of the spacing metric values.

Friedman test can detect the significance of differences between more than two samples [72]. Tables 6 and 7 show the results of Friedman test for the proposed algorithm and the two comparison algorithms. Figs 14 and 15 show the Friedman test chart of the two evaluation metrics. In the figures, the horizontal axis is the average rank value, the vertical axis represents each algorithm. The vertical coordinate 1 represents the proposed algorithm, group 2 represents NSGA-II, and the group 3 represents MOSA. For each algorithm, the dot is used to indicate the average ranking value of that algorithm. Table 6 and Fig 14 compare the HV values, and Table 7 and Fig 15 compare the SP values. It can be seen that the proposed algorithm ranks first in HV, and its mean column rank is significantly different from that of the MOSA. The proposed algorithm performs best in SP, and the mean column rank of the proposed algorithm is obviously different from that of the NSGA-II.

**Table 6:** Friedman test of the HV metric values.

Algorithms	Ranking
Improved OLSR	28(1)
NSGA-II	42(2)
MOSA	50(3)

**Table 7:** Friedman test of the spacing metric values.

Algorithms	Ranking
Improved OLSR	32(1)
NSGA-II	52(3)
MOSA	36(2)

### 6.5 Discussion

Compared to the improvement algorithm proposed in [41] for the OLSR protocol, the above simulation additionally chooses two multi-objective optimization algorithms for comparison, and further increases the persuasiveness by evaluating the quality of the solution set through two metrics, HV and SP, in addition to the metrics used for evaluating the QoS. Compared with the improved algorithm of [42], this simulation adds end-to-end delay to the packet loss rate to compare the improvement effect of the routing protocol, and also proves the reliability of the experimental results by two kinds of non-parametric test.

## 7 Conclusion

This work has worked to provide an effective improvement to OLSR protocol, which integrated an improved mutation operator into NSGA-II framework and combined with the new MPR set selection mechanism. Through simulation and comparison experiments, it can be concluded that the proposed algorithm can effectively reduce the packet loss rate and end-to-end delay. At the same time, this algorithm outperforms other multi-objective optimization algorithms in terms of Pareto solution set evaluation metrics, and the data analysis results also show the superiority of the proposed algorithm. In future work, the multi-objective optimization algorithm will be further optimized in population selection and crossover. Moreover, the algorithm will be applied to more network simulation scenarios to verify its effectiveness.

## Supporting information

S1 File The packet format of OLSR protocol. (PDF)
