# Supplementary material for: A multi-objective optimized OLSR routing protocol
Source: PLoS One. 2024 Apr 26;19(4):e0301842. doi: 10.1371/journal.pone.0301842 (PMC11051643; doi:10.1371/journal.pone.0301842)
Supplement: S1 File — (PDF) [file pone.0301842.s001.pdf]

The packet format of OLSR protocol

The basic layout of any packet in OLSR is as follows (omitting IP and UDP headers):

```
 0                   1                   2                   3
 0 1 2 3 4 5 6 7 8 9 0 1 2 3 4 5 6 7 8 9 0 1 2 3 4 5 6 7 8 9 0 1
+-+-+-+-+-+-+-+-+-+-+-+-+-+-+-+-+-+-+-+-+-+-+-+-+-+-+-+-+-+-+-+-+
|         Packet Length         |    Packet Sequence Number     |
+-+-+-+-+-+-+-+-+-+-+-+-+-+-+-+-+-+-+-+-+-+-+-+-+-+-+-+-+-+-+-+-+
|  Message Type |     Vtime     |         Message Size          |
+-+-+-+-+-+-+-+-+-+-+-+-+-+-+-+-+-+-+-+-+-+-+-+-+-+-+-+-+-+-+-+-+
|                      Originator Address                       |
+-+-+-+-+-+-+-+-+-+-+-+-+-+-+-+-+-+-+-+-+-+-+-+-+-+-+-+-+-+-+-+-+
|  Time To Live |   Hop Count   |    Message Sequence Number    |
+-+-+-+-+-+-+-+-+-+-+-+-+-+-+-+-+-+-+-+-+-+-+-+-+-+-+-+-+-+-+-+-+
|                                                               |
:                            MESSAGE                            :
|                                                               |
+-+-+-+-+-+-+-+-+-+-+-+-+-+-+-+-+-+-+-+-+-+-+-+-+-+-+-+-+-+-+-+-+
|  Message Type |     Vtime     |         Message Size          |
+-+-+-+-+-+-+-+-+-+-+-+-+-+-+-+-+-+-+-+-+-+-+-+-+-+-+-+-+-+-+-+-+
|                      Originator Address                       |
+-+-+-+-+-+-+-+-+-+-+-+-+-+-+-+-+-+-+-+-+-+-+-+-+-+-+-+-+-+-+-+-+
|  Time To Live |   Hop Count   |    Message Sequence Number    |
+-+-+-+-+-+-+-+-+-+-+-+-+-+-+-+-+-+-+-+-+-+-+-+-+-+-+-+-+-+-+-+-+
|                                                               |
:                            MESSAGE                            :
|                                                               |
+-+-+-+-+-+-+-+-+-+-+-+-+-+-+-+-+-+-+-+-+-+-+-+-+-+-+-+-+-+-+-+-+
```
